# Falling corin and ANP activity levels accelerate development of heart failure and cardiac fibrosis

**DOI:** 10.3389/fcvm.2023.1120487

**Published:** 2023-04-18

**Authors:** Inna P. Gladysheva, Ryan D. Sullivan, Guy L. Reed

**Affiliations:** Department of Internal Medicine and Translational Cardiovascular Research Center, College of Medicine, University of Arizona, Phoenix, AZ, United States

**Keywords:** corin, atrial natriuretic peptide, fibrosis, dilated cardiomyopathy, edema

## 1. Introduction

Despite the best available therapies, heart failure with reduced ejection fraction (HFrEF) remains one of the leading causes of morbidity and mortality worldwide (1). Dilated cardiomyopathy (DCM) is one of the leading cause of HFrEF (2). DCM is characterized by progressive heart enlargement with a rEF that is caused by genetic, ischemic, and other disorders (3). Neurohumoral imbalances of the sympathetic nervous system, renin-angiotensin-aldosterone systems (RAAS) and the natriuretic peptide system, are associated with maladaptive cardiac remodeling in HFrEF (4–8). Corin, a cardiac type II transmembrane protease, activates pro-atrial natriuretic peptide (pro-ANP) to biologically active ANP by proteolytic cleavage during pro-ANP secretion from cardiomyocytes (9–12). Through production of biologically active ANP, corin appears to slow the progression of DCM to HFrEF and death, which makes it an attractive therapeutic target in HF management (13–23). Reduced levels of circulating and cardiac corin in patients with symptomatic HFrEF were reported in numerous studies (14, 15, 24–31). The biologically active corin-ANP axis blocked the development of systolic/diastolic dysfunction, low cardiac output, pulmonary and/or systemic fluid retention (edema), dyspnea and elevated blood HF biomarkers (ANP and B-type natriuretic peptide, BNP) (15–18, 23, 30, 31). Pre-clinical studies revealed that the biologically active corin-ANP axis also reduces the development of chronic adverse fibrotic ventricular remodeling (cardiac fibrosis, diffuse accumulation of collagen I/III fibers) (17, 19, 20, 22). Although the protective role of pro-fibrotic angiotensin II (Ang II)-AT1 axis blockage in reverse remodeling in HFrEF is widely accepted, the therapeutic potential of the corin-ANP axis in preventing fibrosis, are less appreciated. Herein, we present and discuss pre-clinical and clinical evidence supporting the targeted restoration of biological activity of the corin-ANP axis as a valuable anti-fibrotic therapeutic strategy in DCM-HFrEF.

## 2. Role of corin-ANP-cGMP pathway under physiological conditions

Under physiological conditions, corin is expressed by atrial and ventricular cardiomyocytes on the external membrane surface as a zymogen and proteolytically active enzyme (9, 10, 17, 21). In atrial cardiomyocytes, corin is co-expressed with its biological substrate pro-ANP- (10, 32). Upon secretion, pro-ANP is proteolytically cleaved by corin and released into circulation as biologically active ANP (11, 12). Circulating biologically active ANP acts locally in the heart and remotely in the kidneys and vasculature by preferentially stimulating the transmembrane natriuretic peptide-A receptor, which generates the intracellular cyclic guanosine monophosphate (cGMP) and stimulates protein kinase G-driven signaling pathways (33, 34). Remotely, the ANP-cGMP axis triggers natriuresis and vasodilation and inhibits renal renin secretion; this decreases cardiac volume overload, aldosterone synthesis and Ang II production in the circulation (11, 35–37). In the heart, the ANP-cGMP pathway counters hypertrophy and fibrosis through autocrine/paracrine regulatory mechanisms leading to inhibition of fibroblast-mediated collagen synthesis (33, 38, 39). Specifically, by stimulation of cGMP production and protein kinase G activation, biologically active ANP may transmit extracellular signals and modulate downstream effector molecules into the same cardiomyocytes it was secreted from (an autocrine mechanism) or on neighboring cardiac myocyte and fibroblast cells (a paracrine mechanism) (33, 39).

## 3. Impairment of corin-ANP-cGMP pathway in symptomatic HFrEF

**Figure 1:**
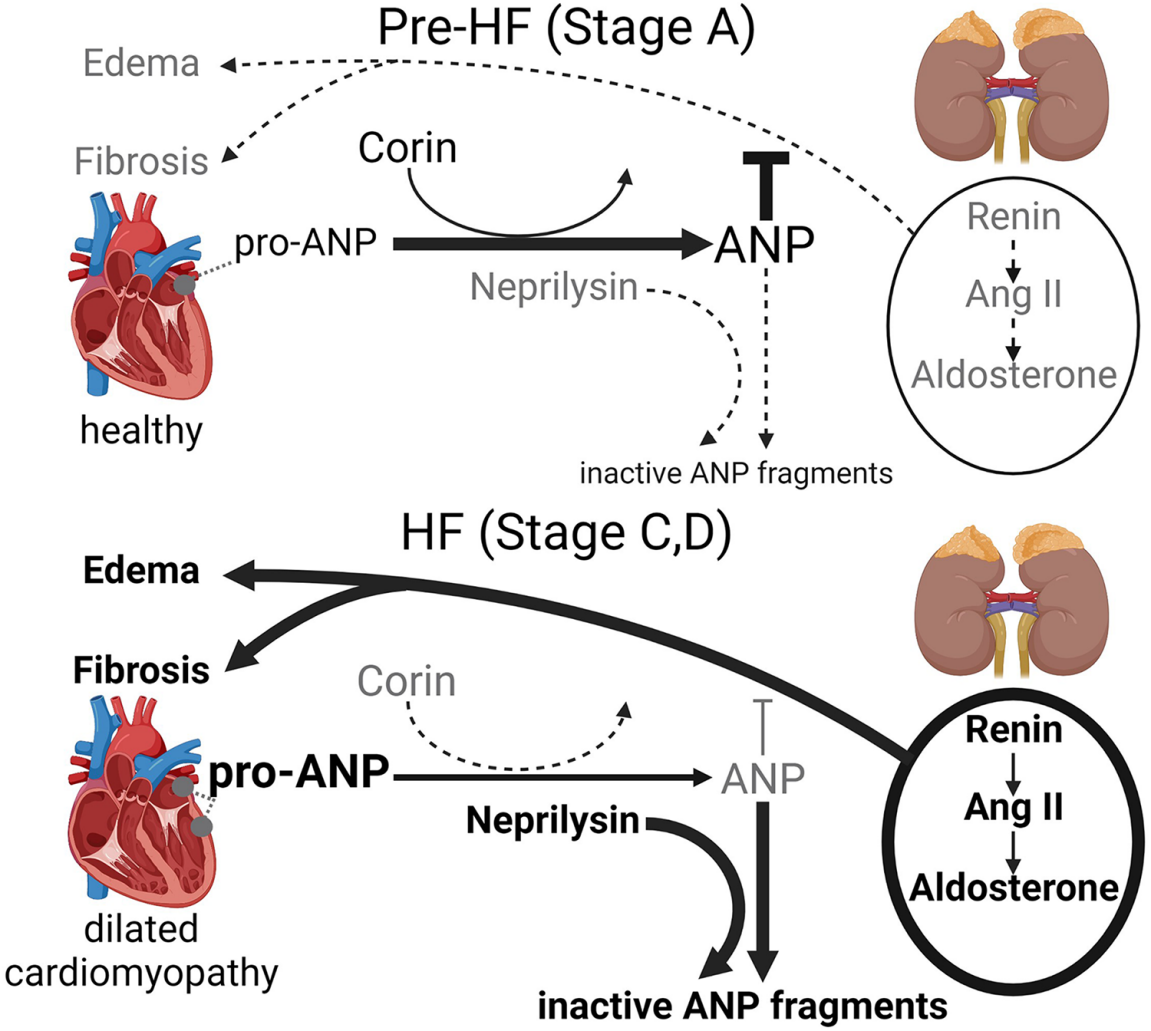
Schematic representation of the pathological shift of the cardiac corin-atrial natriuretic peptide (ANP) axis and classical RAAS [renin-angiotensin II (Ang II)—aldosterone] axis in dilated cardiomyopathy, which promotes cardiac fibrosis, edema and accelerates HFrEF progression.

Dysregulation of ANP-cGMP axis by blunted corin has been shown to contribute to systolic dysfunction, maladaptive cardiac remodeling and edema, leading to HFrEF development (15–19, 21, 22, 30, 40, 41). In DCM, the balance between cardiac anti-fibrotic/pro-fibrotic processes are under control of hemodynamic and humoral modulators such as corin-ANP-cGMP axis and the RAAS. The dysregulation of this balance, its pathological shift and contribution to HFrEF development in DCM are schematically illustrated in Figure 1 and described below.

In DCM at pre-HF stage, RAAS plays an adaptive protective role compensating for impaired cardiac function and structural changes by stimulating sodium-water retention by the kidney and increasing arterial vasoconstriction. However, prolonged, persistent RAAS activation stimulates DCM progression (4–6, 8, 40, 42–44). In DCM at pre-HF stage, the corin-ANP-cGMP axis, when biologically functional, counters the outcomes of the pathologically activated systemic and cardiac classical RAAS by maintaining cardio-renal homeostasis promoting diuresis, natriuresis, and vasodilation and anti-fibrotic action (4, 6, 40, 45, 46). However, as DCM progress in human and mice, cardiac corin expression and activity are reduced leading to impairment of biological activity of the corin-ANP-cGMP axis (21, 30). Declines in corin levels indicate systolic dysfunction as it happened even before the increases in plasma ANP and BNP levels and the onset of edema (21, 23, 26, 30), which is a major hallmark of HF and a key driver of symptoms (3, 47). Consequently, as the natriuretic peptide system is impaired and becomes insufficient to properly balance RAAS activity, pathologically active RAAS further promotes cardiac dilation, fibrotic ventricular remodeling, salt-water retention (edema), and HFrEF development in humans and pre-clinical models (4, 6, 40, 42, 44, 46, 48, 49). Although HFrEF (stages C-D) is associated with a boost of pro-ANP expression by the ventricle's cardiomyocytes (21, 37), pro-ANP cleavage and production of biologically active ANP are compromised as the level of corin is significantly reduced (21).

As DCM progresses to HFrEF (stages C-D), renin is over-secreted by the kidneys into circulation. It triggers Ang II activation pathways (systemic and locally within the heart), cardiac Ang II-independent signaling and stimulates aldosterone secretion from the adrenal glands, which fosters fibrotic remodeling (6, 40, 43, 49, 50). Systemic (circulating) Ang II and aldosterone play an important role in cardiac fibrosis development, as increased local production of Ang II in the heart is not enough to induce ventricular hypertrophy or fibrosis (51).

Converging evidence from human and pre-clinical mouse studies indicate that, as DCM progresses to HF Stages C and D, the protective action of the corin-ANP-cGMP axis is impaired as the coordinated relationship between cardiac pro-ANP expression and enzymes responsible for pro-ANP activation (corin) and ANP degradation (neprilysin) become imbalanced. In particular, levels of the ANP degrading enzyme neprilysin begin to rise (30, 34) while levels of ANP activating enzyme corin fall (14, 15–17, 19, 21– 23, 25, 28, 30, 31). Consequently, the blunted ANP homeostasis contributes to the relative cGMP deficiency in HFrEF.

HFrEF is characterized by elevated pro-ANP expression, which is due to increased expression by the atria and reprogramming of cardiac left ventricular gene expression with induction of pro-ANP. However, levels of cardiac and circulating corin significantly decline in patients and preclinical models with DCM and HFrEF (15, 17, 19, 21, 25, 28, 30, 31, 41). In patients with HFrEF, decreases in circulating corin lead to impaired cleavage/activation of pro-ANP and dysregulated relationships between pro-ANP, ANP and cGMP levels (15, 30). At the same time, neprilysin levels progressively increase with severity of clinical HF assessed by Framingham criteria and are negatively correlated with corin levels (23, 30). In a pre-clinical DCM-HFrEF model, restoration of suppressed cardiac corin was associated with normalization of circulating neprilysin and suppression of renin activity and aldosterone in circulation (41). Low plasma corin was associated with poor HF-related clinical outcomes: lower NYHA functional status (increased functional class), increased cardiovascular mortality and major adverse cardiac events. Depressed cardiac and plasma corin reflects the progression of systolic dysfunction (severity of cardiomyopathy), left ventricular remodeling and fibrosis; it promoted the development of symptomatic HFrEF (17, 21, 30).

## 4. Restoration of corin-ANP-cGMP biological activity protects against cardiac fibrosis and HFrEF development

In experimental DCM, ANP was a critical protective modulator of aldosterone-Ang II-induced interstitial/perivascular fibrosis in the left atrium and ventricle (38). ANP also protected against systolic dysfunction, symptomatic HF, and survival in mice with normal renal function (38). Cardiac pro-ANP deficiency in mice with DCM was associated with significant reduction of cGMP levels in circulation. In these mice, cardiac pro-ANP deficiency was not compensated by cardiac expression of pro-BNP, but was associated with a decline in cardiac transcripts for pro-C-type NP (38), a potent anti-fibrotic modulator that inhibits cardiac fibroblast proliferation and collagen synthesis (34, 38). Consistent with these findings, the survival benefits of neprilysin inhibitors within ARNI therapy (combined Ang II receptor, AT1 and neprilysin inhibitors sacubitril/valsartan) have been attributed in part to its effect on blunting cardiac ventricular remodeling and fibrosis (a risk factor for sudden cardiac death), by preserving biologically active levels of ANP. Thus, ANP circulating levels were elevated after treatment with ARNI therapy, the difference in BNP levels was inconsistent, NT-pro-BNP levels decreased and CNP levels were not affected by treatment (34, 52–54). Increases in ANP plasma levels in patients with ARNI therapy for chronic HFrEF were associated with increased urinary cGMP levels (55). Another study demonstrated that in patients with acute decompensated HFrEF, ARNI therapy was associated with higher urinary cGMP levels (56). However, in both these studies (55, 56), corin levels were not analyzed.

Similar to ANP, genetic restoration of both proteolytically active or inactive cardiac corin in mice with DCM improved systolic function, delayed symptomatic HFrEF progression and prolonged survival (17, 18, 41). However, only proteolytically active (ANP-cleaving) cardiac corin has protective anti-fibrotic action (17, 41). Cardiac restoration of proteolytically active corin led to a significant reduction in cardiac collagen I/III transcripts and a trend towards reduction of TGF*β* transcripts, and overall suppression of interstitial and perivascular ventricular fibrosis (17). Restoration of cardiac corin significantly increased pro-ANP cleavage to ANP and cGMP production, both of which are potent inhibitors of cardiac fibroblast proliferation and collagen synthesis (17). Cardiac-specific overexpression of proteolytically active corin reduced myocardial infarct size 24 h post-experimental myocardial infarction (MI) induced by left coronary artery ligation in mice. Corin overexpression prevented these mice from development of severe systolic dysfunction, cardiac remodeling and edema 4 weeks post-MI (57). However, this study did not assess the impact of cardiac corin overexpression on the pro-ANP-cGMP axis and cardiac fibrosis. In mouse HF models induced by left coronary artery ligation and transverse aortic constriction, intraperitoneal injection of a recombinant extracellular fragment of human corin with an engineered activation site lowered Ang II and aldosterone plasma levels, boosted cGMP levels, improved cardiac function and attenuated cardiac remodeling and fibrosis (22). The analysis of pro-ANP metabolism in the plasma of patients with stable chronic HFrEF, indicated that ARNI therapy increased pro-ANP cleavage, which was linked to an increase in corin activity (58).

Considering the above knowledge, we hypothesize that enhancing cardiac corin expression by ARNI therapy might contribute to improved cardiac remodeling in HFrEF. Thus, ARNI therapy could provide beneficial antifibrotic outcomes by suppressing the profibrotic action of angiotensin II and boosting antifibrotic ANP activity. Increased ANP activity may be achieved not only through reduced degradation of biologically active ANP by neprilysin, but also through a feedback mechanism of improved systolic function stimulating cardiac corin expression, which in turn improves pro-ANP cleavage and increases biologically active ANP levels. It is worth testing the hypothesis that in HFrEF patients, ARNI therapy is associated with increased corin levels in circulation and cardiac left ventricle and reduced impairment of pro-ANP cleavage, which contribute to reverse cardiac remodeling.

## 5. Conclusions and translational value

Available experimental and clinical evidence suggests that in DCM, dysregulation of the biological effects of ANP, at least in part by insufficient corin expression and/or activity, promotes cardiac fibrosis associated with relative cGMP deficiency and contributes to the progression of systolic dysfunction and symptomatic HFrEF. These insights may suggest a new therapeutic paradigm to prevent DCM from becoming a relentless, progressive and fatal form of HFrEF. Preserving or boosting the biological activity of the corin-ANP-cGMP axis by corin targeted interventions may offer potential therapeutic strategies for preventing or blocking progressive cardiac fibrosis in DCM-HFrEF.
